# A double-blind, randomized clinical trial of dietary supplementation on cognitive and immune functioning in healthy older adults

**DOI:** 10.1186/1472-6882-14-43

**Published:** 2014-02-04

**Authors:** John E Lewis, Angelica B Melillo, Eduard Tiozzo, Lawrence Chen, Susanna Leonard, Mark Howell, Janelle Diaz, Kathy Gonzalez, Judi M Woolger, Janet Konefal, Elaine Paterson, David Barnes

**Affiliations:** 1 Department of Psychiatry & Behavioral Sciences, University of Miami Miller School of Medicine, 1120 NW 14th Street, Miami, FL 33136, USA; 2 Medical Wellness Center, University of Miami Miller School of Medicine, 1120 NW 14th Street, Miami, FL 33136, USA; 3 Department of Medicine, University of Miami Miller School of Medicine, 1120 NW 14th Street, Miami, FL 33136, USA; 4 Standard Process, 1200 W. Royal Lee Drive, Palmyra, WI 53156, USA

**Keywords:** Cognitive functioning, Immune functioning, Ginkgo biloba, Choline, Dietary supplement, Older adults, Trail making test, Controlled oral word association, Epidermal growth factor

## Abstract

**Background:**

Declining cognitive function is relatively common and increasingly prevalent. Studies have shown that different nutrients (e.g., Ginkgo biloba and vitamin E) appear to be effective at improving memory and concentration, while less is known about their effect on immunity.

**Methods:**

This study investigated the effect of Ginkgo Synergy® plus Choline (n = 33) and OPC Synergy® plus Catalyn® (n = 31) versus placebo (n = 33) in a 6-month, randomized, double-blind trial on cognitive and immune functioning among English-speaking, non-smoking, healthy older adults. The Stroop Color and Word Test, Trail Making Test A and B, Controlled Oral Word Association, Hopkins Verbal Learning, Mini-Mental State Exam, and Digit Symbol were administered at baseline and 3 and 6 months follow-up to assess cognitive functioning. Cytokines and growth factors were measured at baseline and 6 months to assess inflammation and immune functioning. Data were analyzed with linear mixed modeling.

**Results:**

No serious adverse events were noted in this study. According to time on the Trail Making Test-B, the Ginkgo Synergy® plus Choline arm showed improvement from baseline to 3 months follow-up (mean difference = 24.2; SE = 6.4; 95% CI: 8.6, 39.7; p = 0.01). On the Controlled Oral Word Association Trial-S, the scores significantly increased for the Ginkgo Synergy® plus Choline arm from baseline to 6 months follow-up (mean difference = 2.1; SE = 0.8; 95% CI: 0.2, 3.9; p < 0.05) and for the OPC Synergy® plus Catalyn® arm from baseline to 3 months follow-up (mean difference = 2.1; SE = 0.8; 95% CI: 0.2, 4.0; p < 0.05). Epidermal growth factor significantly decreased from baseline to 6 months follow-up for the Ginkgo Synergy® plus Choline arm (mean difference = 120.7; SE = 28.4; 95% CI: 62.6, 178.8; p < 0.001).

**Conclusions:**

Our study showed isolated and modest effects of a Ginkgo biloba plus choline-based formula on cognitive and immune functioning among healthy older adults with no history of significant cognitive deficits. Our trial was registered with clinicaltrials.gov (ID: NCT01672359). This study was supported by a grant from Standard Process, Inc.

## Background

Approximately 5 million Americans suffer from some form of dementia, the leading cause of institutionalization among the elderly [1]. Alzheimer’s disease (AD), the most common form of dementia, accounts for over two-thirds of the cases. The number of people over the age of 65 will triple and those over the age of 85 will quadruple in the next 50 years, and 14 million Americans will be diagnosed with AD by the middle of the 21st century [2].

Conventional Western medicine cannot prevent cognitive decline, leaving many consumers turning to alternative treatments, such as nutritional therapies [2]. Total sales of dietary supplements, including those touted to enhance cognition, were estimated at over $30 billion in 2010 [3]. Among males and females 60 and older, the rate of dietary supplement use was higher, compared to males and females between 20 and 59 (42% and 54% vs. 35% and 47%, respectively) [4]. Many herbs are also consumed with inconsistencies in usage attributed to variation in the definition of herbs and the length of use [5].

Several clinical trials have utilized Ginkgo biloba to assess the effect of dietary supplements on cognition [6-8]. A meta-analysis among studies of AD patients with Ginkgo biloba revealed a small effect size of 0.40 (p < 0.0001) on cognitive function [9]. Other nutrients may hold promise as well, e.g., pre-clinical data have showed that grape seed may be effective for preventing cognitive decline, while decreasing β-amyloid proteins in the brain [10]. Additionally, aging is accompanied by systemic inflammation, and cytokines have been associated with cognitive decline in older individuals [11,12]. However, the relationship between cytokine-mediated physiological processes with aging and cognitive decline remains poorly understood.

Our study extends the evaluative process of nutritional therapies through a randomized, double-blind, placebo-controlled clinical trial assessing a regimen of dietary supplements’ efficacy. We hypothesized that two dietary supplement formulae consisting of Ginkgo Synergy® plus Choline or OPC Synergy® plus Catalyn® would result in improved cognitive and immune functioning compared to placebo in a sample of healthy older individuals.

## Methods

### Study participants

**Figure 1:**
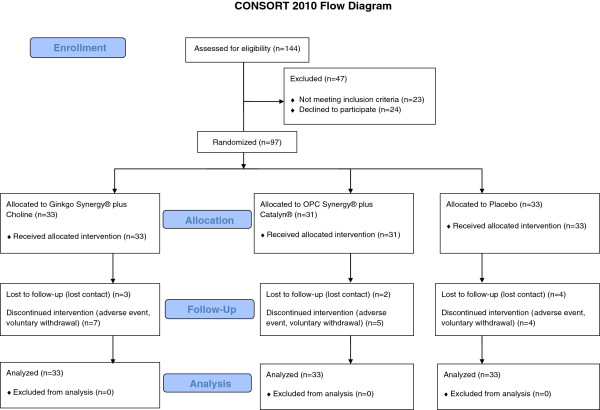
The CONSORT flowchart.

The study was conducted with the approval of the University of Miami Institutional Review Board for human subjects research. Each subject signed informed consent and HIPAA forms prior to study entry. Potential participants (n = 144) were identified through referrals from offices at the University of Miami Miller School of Medicine and community centers in Miami-Dade, Broward, and Palm Beach counties during May 2010 to December 2011. Out of 121 eligible participants, 97 were enrolled (Ginkgo Synergy® plus Choline, n = 33; OPC Synergy® plus Catalyn®, n = 31; and placebo, n = 33). Please see Figure 1 for the CONSORT flowchart.

### Study design

Participants were randomized into a three-group, parallel, placebo-controlled clinical trial to assess the efficacy of two dietary supplement formulae on cognitive and immune functioning in healthy older adults.

#### Inclusion and exclusion criteria

Inclusion criteria were: (a) 60+ years of age; (b) English speaking; (c) not living in a nursing facility; (d) no use of dietary supplements for cognitive functioning two weeks before enrolling and during the intervention period; and (e) a Mini-Mental State Exam (MMSE) score ≥23. Exclusion criteria were: (a) AD or related disorders; (b) schizophrenia, psychotic disorders, bipolar, major depression with psychotic features, or delirium; (c) bleeding disorders; (d) aphasia or sensory, motor, or visual disturbances that would have interfered with testing; (e) cancer, cardiovascular, pulmonary, renal, thyroid, hepatic, gastrointestinal diseases, or insulin-dependent diabetes; (f) current cigarette smoking or alcohol or substance abuse/dependence; (g) 3+ major medical or psychiatric hospitalizations in the past year; (h) a T score >70 on the Global Severity Index of the Brief Symptoms Inventory (BSI); (i) a score ≥29 on the Beck Depression Inventory-II (BDI); (j) prescription or OTC sympathomimetic amines and antihistamines within 2 days of an assessment visit; (k) cognition-enhancing drugs; or (l) Coumadin, tricyclic antidepressants, antipsychotics, or anticonvulsants.

#### Screening

Eligible participants completed the Short Portable Mental Status Questionnaire (SPMSQ) and the Wechsler Memory Scale III Story A (WMS-III-A). Subjects were allowed only two errors on the SPMSQ. If they scored <6 points on story A for the WMS-III-A, they were given a second opportunity and had to score a 4+ on story B. If all pre-screening criteria were achieved, subjects were scheduled for the additional screening (MMSE, BSI, and BDI) and the baseline assessment. Mental status was assessed with the MMSE, providing a rapid screen of orientation, registration, attention and calculation, recall, and language domains. As psychological distress and depression may impair cognitive function, and thus lead to poor performance on outcome measures, participants were also evaluated using the BSI and BDI.

#### Intervention and randomization

After screening and baseline, participants were randomly assigned to one of three conditions: (a) Ginkgo Synergy® (2 capsules/day providing 120 mg/day Ginkgo biloba leaf, 80 mg/day Ginkgo biloba whole extract, 40 mg/day grape seed extract, Gotu kola leaf (*Centella asiatica*), dried buckwheat leaf juice, buckwheat seed, and soybean lecithin powder) plus Choline (4 tablets/day providing 700 mg/day), (b) OPC Synergy® (2 capsules/day providing 100 mg/day grape seed extract, 50 mg/day green tea extract (60% catechins), 50 mg/day bilberry fruit (25% anthocyanins), dried buckwheat leaf and juice, green tea leaf powder, and dried carrot root) plus Catalyn® (4 tablets/day providing 312 IU/day vitamin D, 1,600 IU/day vitamin A, 5.3 mg/day vitamin C, 0.3 mg/day thiamine, 0.3 mg/day riboflavin, 1.3 mg/day vitamin B6, defatted wheat germ, carrot (root), calcium lactate, nutritional yeast, bovine adrenal, bovine liver, magnesium citrate, bovine spleen, ovine spleen, bovine kidney, dried pea (vine) juice, dried alfalfa (whole plant) juice, mushroom, oat flour, soybean lecithin, and rice bran), or (c) placebo (cellulose, lactose, and beet powder) provided by the manufacturer (Standard Process, Palmyra, WI). The table of random permutations was created by one of the authors (JEL). It was created in blocks of 10 subjects at a time, and study coordinators assigned participants to an intervention arm in sequential fashion. All subjects and investigators were blind to treatment condition and remained blinded until after data analysis; only a staff member at Standard Process knew the assignment.

#### Outcomes and assessments

All participants completed a basic sociodemographics and medical history questionnaire and reported their list of medications at baseline.

#### Cognitive measures

The neuropsychological assessment targeted executive functioning, memory, and psychomotor speed and was administered at baseline and 3 and 6 months follow-up. Measures included the MMSE, the Stroop Color and Word Test (SCWT), the Trail Making Test Parts A and B (TMT-A, TMT-B), the Controlled Oral Word Association test (COWA), the Digit Symbol subtest of the Wechsler Adult Intelligence Scale, Third Edition (WAIS-III), and the Hopkins Verbal Learning Test-Revised (HVLT-R).

The SCWT assesses response inhibition [13], the effects of perceptual interference [14], concentration, and speed of processing abilities, involving three distinct tasks. TMT-A and TMT-B assess a person’s abilities to sequence and shift perceptual sets, concentration and vigilance, and visuomotor scanning and tracking speed [15], which reflect executive control functioning [13]. These instruments are exceptional measures of general brain functioning [16]. The COWA assesses verbal fluency and is sensitive to cerebral dysfunction [17]. The WAIS-III Digit Symbol subtest measures perceptual speed, during which the subject must quickly copy symbols that are linked with numbers [18]. The HVLT-R assesses verbal and visuospatial recall and recognition memory, containing two equivalent forms to allow repeat testing without correction for practice effects [19].

The administration of the battery required 60-90 minutes. Participants were provided at least one break during the testing session and were closely monitored for fatigue or emotional distress. No participants suffered from excessive fatigue to the point of needing to complete the assessment on a later date.

#### Immune function markers

Venous blood was obtained at baseline and 6 months. Blood samples were collected in EDTA tubes and frozen within 2 hours of collection. Samples were sent to a contract lab (Radix Biosolutions, Georgetown, TX) for analysis. A commercially-available magnetic bead-based Milliplex® MAG kit (Millipore Corporation MA) was used to analyze all samples for: interleukin (IL)-2, IL-4, IL-6, IL-8, IL-10, IL-1α, IL-1β, interferon (IFN)-γ, tumor necrosis factor (TNF)-α, monocyte chemotactic protein (MCP)-1, vascular endothelial growth factor (VEGF), and epidermal growth factor (EGF). The kit included two quality control samples, expected concentrations for each sample, and all necessary reagents. The standard curve and quality controls were prepared fresh each day. Both quality control samples were run in duplicate before and after each set of samples on each microtiter plate to produce 2 concentration values per quality control sample in each assay. Assays were set up sequentially, so all samples could be analyzed on the same day, utilizing five separate assay kits. Each sample was run neat in duplicate wells and incubated at 4°C overnight. Sample reruns were completed with the original samples and involved an additional freeze/thaw cycle. The concentration of each cytokine or growth factor in each plasma specimen was calculated from the standard curve and reported in pg/ml.

#### Intervention protocol

Supplements were given to each participant at the baseline and 3-month follow-up assessments to promote greater compliance. Subjects were not advised to modify eating or physical activity habits or prescription medication use. In addition, they were instructed not to consume other dietary supplements containing Ginkgo biloba, vitamin B complex nutrients, vitamin E, or any other cognitive-enhancing nutritional supplement. Subjects listed all dietary supplements taken on the health history form, and products were reviewed to ensure none of these nutrients was consumed during the course of the trial. Regardless of study arm assignment, subjects consumed 3 tablets 2 times per day with breakfast and dinner, i.e., 6 tablets per day total. Each subject was compensated $35 per assessment at baseline and 3 and 6 months follow-up.

#### Statistical analysis

Data were analyzed using SPSS 19 (IBM Inc., Chicago, IL) for Windows. Frequency and descriptive statistics were calculated on all variables. Analysis of variance and chi square were utilized to determine the presence of differences in background contextual variables by study arm assignment. We utilized linear mixed modeling (LMM) to assess the fixed effect of time by randomization on changes in our outcome variables from baseline to 6 months follow-up. If the type III test of the fixed effect of time by randomization was significant, then we used pairwise comparisons to determine the unique differences in effects over time by study arm between baseline and follow-up at 3 and 6 months for the cognitive assessment and between baseline and 6 months for the physiological variables. LMM with heterogeneous compound symmetry covariance allowed us to account for subject attrition, inter-correlated responses between time points, and non-constant variability. Based on the results that Mix and Crews found in response to a 6-week placebo-controlled trial of Ginkgo biloba on the SCWT color-naming task [7], we estimated an effect size of 5.25 units on the task, which gave us power of roughly 80% with 31 subjects in each group. The criterion for statistical significance was α = 0.05.

## Results

### Safety and tolerability

Three subjects withdrew from the study: (a) one placebo group subject reported insomnia and heightened energy at night; (b) one OPC Synergy® plus Catalyn® group subject was diagnosed with ulcerative colitis shortly after enrolling in the study; and (c) one Ginkgo Synergy® plus Choline group subject reported joint aches that were not alleviated by titrating the dose by 1/3 and then increasing the dose by 1/3 every 3 days. No other adverse events were reported.

### Sociodemographics, health risk, and medication use

Table 1 presents sociodemographics by study arm. Only race/ethnicity showed a statistically significant difference, as more Hispanics were assigned to the Ginkgo Synergy® plus Choline study arm (*X*^2^(4) = 14.2, p < 0.01). Table 2 displays the prevalence of current prescription medications and OTC remedies. A higher percentage of subjects in the OPC Synergy® plus Catalyn® study arm was taking some type of medication for dyslipidemia or high cholesterol (*X*^2^(2) = 6.3, p = 0.04). No other proportion was significantly different.

**Table 1:** Sociodemographic characteristics of the sample

**Variable**	**Category**	**Total sample (n = 97)**	**(OPC Synergy® + Catalyn®) (n = 31)**	**(Ginkgo Synergy® + Choline) (n = 33)**	**Placebo (n = 33)**	**Statistic**
Age	-	M = 68.8 (SD = 7.2; R = 58, 93)	M = 68.5 (SD = 6.7; R = 59, 83)	M = 67.6 (SD = 6.3; R = 58, 82)	M = 70.3 (SD = 8.3; R = 60, 93)	F(2,96) = 1.2, p = 0.30
Gender	Male	27 (27.8%)	7 (22.6%)	8 (24.2%)	12 (36.4%)	*X*^2^(2) = 1.3, p = 0.40
	Female	70 (72.2%)	24 (77.4%)	25 (75.8%)	21 (63.6%)	
Race/ethnicity	White, non-Hispanic	80 (82.5%)	27 (87.1%)	21 (63.6%)	32 (97.0%)	*X*^2^(4) = 14.2, p < 0.01
	Black, non-Hispanic	2 (2.1%)	1 (3.2%)	1 (3.0%)	-	
	Hispanic	15 (15.5%)	3 (9.7%)	11 (33.3%)	1 (3.0%)	
Education	Up to high school	12 (12.4%)	5 (16.1%)	5 (15.2%)	2 (6.1%)	*X*^2^(6) = 11.4, p = 0.08
	Some post high school training	34 (35.1%)	11 (35.5%)	12 (36.4%)	11 (33.3%)	
	College graduate	24 (24.7%)	7 (22.6%)	12 (36.4)	5 (15.2%)	
	Master’s degree or higher	27 (27.8%)	8 (25.8%)	4 (12.1%)	15 (45.5%)	
Marital status	Never married	8 (8.2%)	-	2 (6.1%)	6 (18.2%)	*X*^2^(6) = 9.7, p = 0.14
	married	49 (50.5%)	16 (51.6%)	15 (45.5%)	18 (54.5%)	
	widowed	19 (19.6%)	8 (25.8%)	7 (21.2%)	4 (12.1%)	
	Divorced/separated	21 (21.6%)	7 (22.6%)	9 (27.3%)	5 (15.2%)	

Note: M = Mean; SD = Standard Deviation; R = Range.

**Table 2:** Prevalence of prescription and over-the-counter medication usage

**Medication**		**Category**	**Total sample (n = 97)**	**(OPC Synergy® + Catalyn®) (n = 31)**	**(Ginkgo Synergy® + Choline) (n = 33)**	**Placebo (n = 33)**	**Statistic**
Current prescription	Anti-depressant	Yes	11 (11.3%)	6 (19.4%)	1 (3%)	4 (12.1%)	*X*^2^(2) = 4.3, p = 0.12
		No	86 (88.7%)	25 (80.6%)	32 (97%)	29 (87.9%)	
	Beta blocker	Yes	7 (7.2%)	4 (12.9%)	3 (9.1%)	-	*X*^2^(2) = 4.2, p = 0.12
		No	90 (92.8%)	27 (87.1%)	30 (90.1%)	33 (100%)	
	Anti-hypertensive	Yes	25 (25.8%)	9 (29%)	9 (27.3%)	7 (21.2%)	*X*^2^(2) = 0.6, p = 0.75
		No	72 (74.2%)	22 (71%)	24 (72.2%)	26 (78.8%)	
	Anti-hypercholesterol	Yes	11 (11.3%)	7 (22.6%)	1 (3%)	3 (9.1%)	*X*^2^(2) = 6.3, p = 0.04
		No	86 (88.7%)	19 (77.4%)	32 (97%)	30 (90.1%)	
	Laxative	Yes	7 (7.2%)	2 (6.5%)	3 (9.1%)	2 (6.1%)	*X*^2^(2) = 0.3, p = 0.88
		No	90 (92.8%)	29 (93.5%)	30 (90.1%)	31 (93.9%)	
	Insomnia	Yes	10 (10.3%)	3 (9.7%)	4 (12.1%)	3 (9.1%)	*X*^2^(2) = 0.2, p = 0.91
		No	87 (89.7%)	28 (90.3%)	29 (87.9%)	30 (90.1%)	
OTC in the prior week	Aspirin	Yes	33 (34%)	10 (32.3%)	14 (42.4%)	9 (27.3%)	*X*^2^(2) = 1.8, p = 0.42
		No	64 (66%)	21 (67.7%)	19 (57.6%)	24 (72.2%)	
	Tylenol	Yes	34 (35.1%)	12 (38.7%)	13 (39.4%)	9 (27.3%)	*X*^2^(2) = 1.3, p = 0.51
		No	63 (64.9%)	19 (61.3%)	20 (60.6%)	24 (72.2%)	
	Antacid	Yes	13 (13.4%)	6 (19.4%)	5 (15.2%)	2 (6.1%)	*X*^2^(2) = 2.6, p = 0.28
		No	84 (86.6%)	25 (80.6%)	28 (84.8%)	31 (93.9%)	
	Vitamin/mineral	Yes	67 (69.1%)	21 (67.7%)	25 (75.8%)	21 (63.6%)	*X*^2^(2) = 1.2, p = 0.56
		No	30 (30.9%)	10 (32.3%)	8 (24.2%)	12 (36.4%)	

### Cognitive functioning

We found no significant fixed effects for time, randomization, or time by randomization for the Word T, Color T, Color-Word T, Predicted Color-Word, or Interference T on the SCWT or for the MMSE.

**Figure 2:**
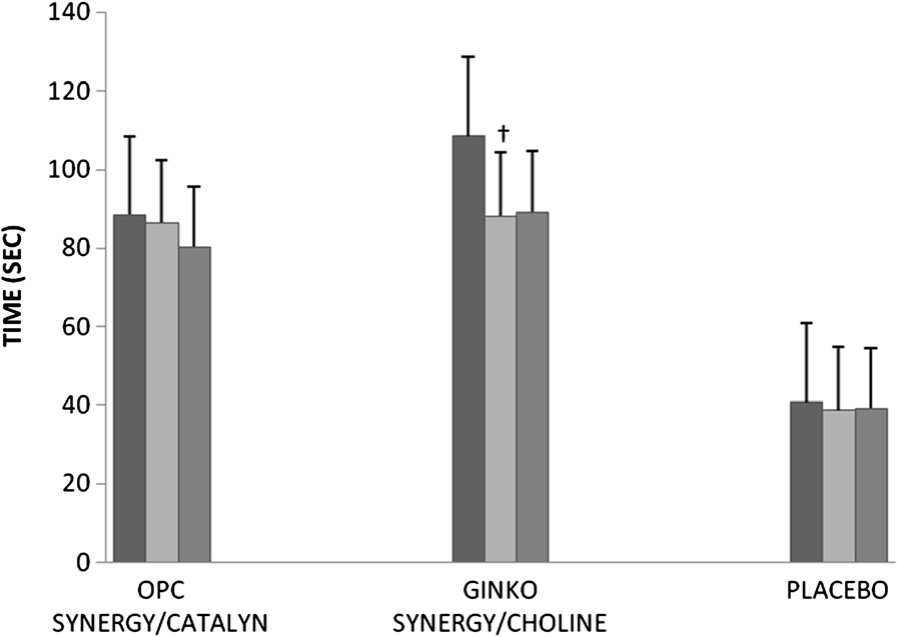
**Trail Making Test Part B at baseline, 3 months, and 6 months.** Baseline = Black, 3 months follow-up = light gray, 6 months follow-up = dark gray; †Significantly different from baseline within the same group (p = 0.01).

For time and errors on the TMT-A, the fixed effects for time, randomization, and time by randomization were non-significant. For time on the TMT-B (see Figure 2), the fixed effect for time by randomization was significant (F[4,113.8] = 2.76, p < 0.05). Time significantly decreased from baseline to 3 months (mean difference = 24.2; SE = 6.4; 95% CI: 8.6, 39.7; p = 0.01), but not at 6 months (mean difference = 14.6; SE = 7.1; 95% CI: -2.7, 32.0; p = 0.12) for the Ginkgo Synergy® plus Choline study arm.

The Digit Symbol test included number correct, number incorrect, total score, and total proportion. For the number correct, the fixed effect for time was significant (F[2,139.1] = 6.6, p < 0.01), but the effects for randomization and time by randomization were non-significant. For the number incorrect, the fixed effect for time was significant (F[2,133.9] = 5.7, p < 0.01), but the effects for randomization and time by randomization were non-significant. For the total score, the fixed effect for time was significant (F[2,137.3] = 4.2, p < 0.05), but the effects for randomization and time by randomization were non-significant. For the total proportion, the fixed effect for time was significant (F[2,139.0] = 5.3, p < 0.01), but the effects for randomization and time by randomization were non-significant.

The results for the HVLT-R included total recall T score, delayed recall T score, retention T score, and recognition discrimination index T score. For the total recall T score, the fixed effect for time was significant (F[2,100.4] = 10.4, p < 0.001), but the effects for randomization and time by randomization were non-significant. For the delayed recall T score, the fixed effect for time was significant (F[2,128.8] = 6.2, p < 0.01), but the effects for randomization and time by randomization were non-significant. For the retention T score and the recognition discrimination index T score, no significant fixed effects were found.

**Figure 3:**
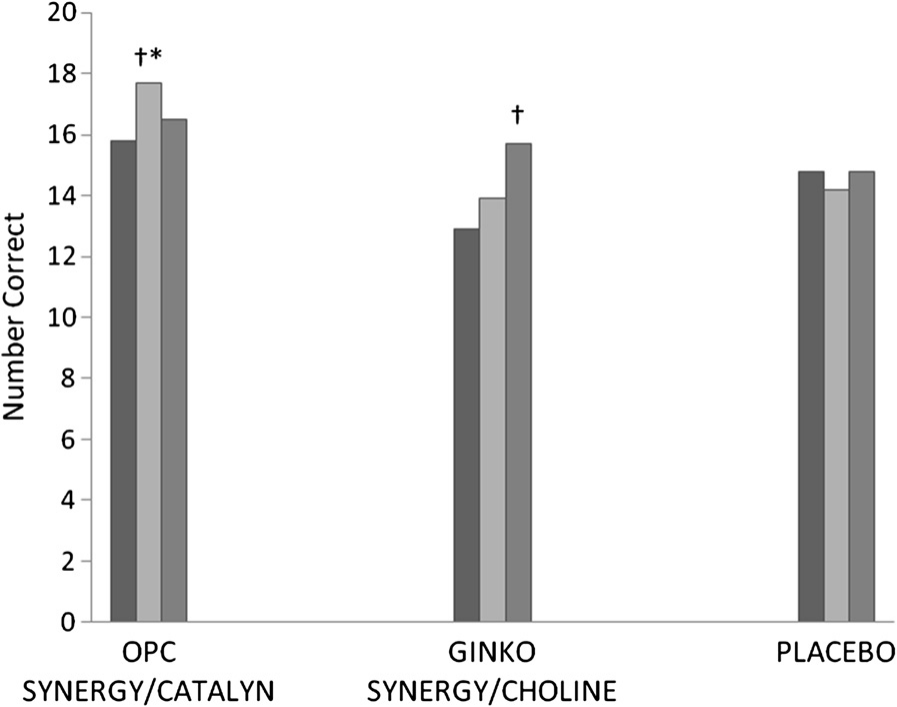
**Controlled Oral Word Association Trial 3-S test at baseline, 3 months, and 6 months.** Baseline = Black, 3 months follow-up = light gray, 6 months follow-up = dark gray; †Significantly different from baseline within the same group (p = 0.05); *Significantly different from Ginkgo Synergy/Choline and placebo at the same time point (p = 0.05).

For the COWA test, the fixed effect for time was significant (F[2,124.5] = 7.3, p < 0.01), but the effects for randomization and time by randomization were non-significant on Trial 1-F. For Trial 2-A, the fixed effect for time was significant (F[2,125.9] = 6.4, p < 0.05), but the effects for randomization and time by randomization were non-significant. For Trial 3-S (see Figure 3), the fixed effects for time (F[2,129.8] = 4.1, p < 0.05) and randomization (F[2,96.1] = 3.1, p = 0.05) were significant, and the time by randomization effect was marginally significant (F[4,129.9] = 2.2, p = 0.07). Post-hoc comparisons revealed that the score at 3 months follow-up of the OPC Synergy® plus Catalyn® study arm was significantly higher than the Ginkgo Synergy® plus Choline (mean difference = 3.9; SE = 1.4; 95% CI: 0.5, 7.3; p < 0.05) and placebo (mean difference = 3.6; SE = 1.4; 95% CI: 0.3, 7.0; p < 0.05) study arms. The score for the OPC Synergy® plus Catalyn® study arm significantly increased from baseline to 3 months follow-up (mean difference = 2.1; SE = 0.8; 95% CI: 0.2, 4.0; p < 0.05), but did not continue increasing at 6 months (mean difference = 1.0; SE = 0.8; 95% CI: -0.8, 2.9; p = 0.47). For the Ginkgo Synergy® plus Choline study arm, the score significantly increased from baseline to 6 months follow-up (mean difference = 2.1; SE = 0.8; 95% CI: 0.2, 3.9; p < 0.05). The placebo group showed no significant changes from baseline to 6 months follow-up. For Trials 1-3 Total, the fixed effect for time (F[2,130.7] = 13.0, p < 0.001) was significant, but the effects for randomization and time by randomization were non-significant.

### Immune functioning

**Figure 4:**
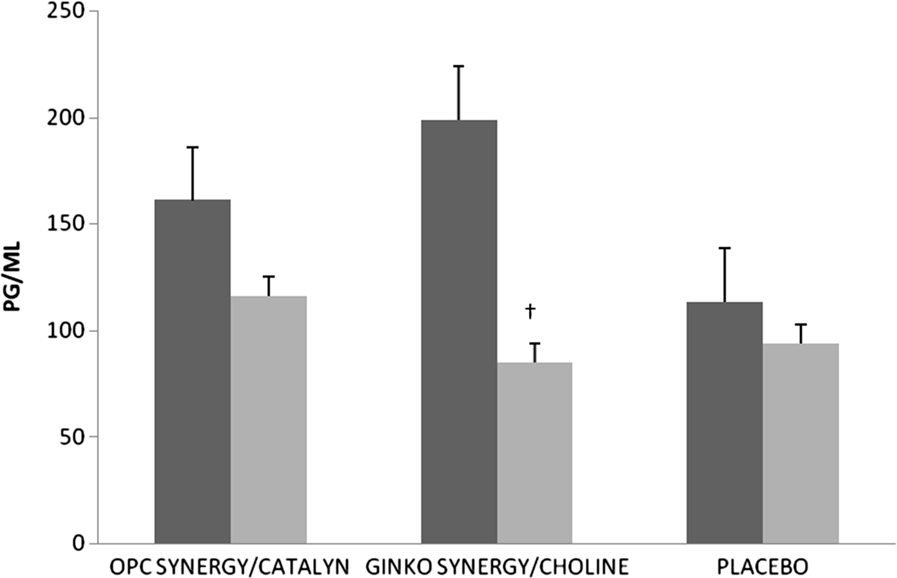
**Epidermal growth factor at baseline and 6 months.** Baseline = Black and 6 months follow-up = gray; †Significantly different from baseline within the same group (p = 0.001).

IL-4 was completely undetectable by the assay, other than for 2 values at baseline. For IL-2, IL-6, IL-8, IL-10, IL-1α, IFN-γ, TNF-α, and MCP-1, the fixed effects for time, randomization, and time by randomization were non-significant. For IL-1β, the fixed effect for time (F[1,12.6] = 6.3, p < 0.05) was significant, but the effects for randomization and time by randomization were non-significant. For VEGF, the fixed effect for time (F[1,41.2] = 5.9, p < 0.05) was significant, but the effects for randomization and time by randomization were non-significant. For EGF, the time by randomization effect was marginally significant (F[2,28.7] = 2.8, p = 0.08). Post-hoc comparisons revealed that EGF for the Ginkgo Synergy® plus Choline study arm significantly decreased from baseline to 6 months follow-up (mean difference = 120.7; SE = 28.4; 95% CI: 62.6, 178.8; p < 0.001). EGF did not significantly change for the OPC Synergy® plus Catalyn® and placebo arms (see Figure 4).

## Discussion

In the current study, we demonstrated several significant and isolated improvements with two dietary supplement formulae of Ginkgo Synergy® plus Choline and OPC Synergy® plus Catalyn. The Ginkgo Synergy® plus Choline study arm showed improvements after 3 months on the TMT-B time score (68%) and after 6 months on the COWA Trial-S score (18%). Globally, the TMT is a valid and reliable assessment used to detect impairment in many cognitive areas [20]. The TMT-B has been shown to be a valid indicator of executive functioning [21] and is likely to be more sensitive to assessing cognitive flexibility [20]. The OPC Synergy® plus Catalyn® study arm showed an 11% improvement in the COWA Trial-S score at 3 months follow-up. The COWA assesses the ability to make verbal associations to certain letters (in this case, “S”) [22]. It has been used as a key part of neuropsychological testing to rule out common cognitive disorders, and it has been associated with frontal lobe lesions [23] and AD [24].

Our findings are similar to other studies that showed improvements with Ginkgo biloba-based formulae in certain aspects of cognitive functioning in healthy adults [7,8] and among participants with probable AD [25]. Mix and Crews investigated the 6-week effect of Ginkgo biloba extract (180 mg/day of EGb 761) compared to placebo on cognitive functioning in 48 healthy elderly (55 years of age and above) participants [7]. The participants on Ginkgo biloba extract significantly improved on the SCWT (i.e., a task assessing speed of processing abilities) at follow-up compared to participants on placebo, and non-significant trends were noted on three of the four remaining cognitive tasks involving a timed, processing component. Additionally, more participants in the Ginkgo biloba group rated their ability to remember at follow-up as “improved” compared to the placebo group. Napryeyenko and colleagues analyzed the data of 395 participants (≥50 years of age) with dementia (214 with probable AD or possible AD with cerebrovascular disease and 181 with probable vascular dementia) to assess the effect of 22 weeks of a Ginkgo biloba extract (EGb 761® 240 mg/day) versus placebo on the Short Syndrome Test (SKT), a cross-culturally validated cognitive test battery, the Neuropsychiatric Inventory, the Verbal Fluency Test, the Clock-Drawing Test, the Hamilton Rating Scale for Depression, and the Gottfries-Bråne-Steen Scale [25]. Those with AD and vascular dementia taking the Ginkgo biloba extract had higher SKT total scores by -3.0 ± 2.3 and -3.4 ± 2.3, respectively, compared to the participants on placebo whose scores decreased by +1.2 ± 2.5 and +1.5 ± 2.2 points, respectively (p < 0.01 for each drug-placebo difference). Significant drug-placebo differences were noted for all other outcome variables with no differences between AD and vascular dementia subgroups. Adverse events were higher for the placebo group. Conversely, another study failed to show any improvements with Ginkgo biloba on memory and related mental functions among older adults [6]. Solomon et al. investigated the effect of 40 mg per day of Ginkgo biloba compared to placebo on cognitive functioning for 6 weeks among 230 community-dwelling healthy participants 60 years of age and older. The results of the study did not result in any improvements in learning, memory, attention, or concentration between the two groups at the follow-up assessment [6]. Inconsistencies in the findings of our study versus others can be due to dose, plant form, extract strength, and other associated production and quality factors.

Our study may be one of the first of its kind to assess a panel of cytokines and growth factors before and after dietary supplementation in healthy older adults. To our knowledge, cytokines have been assessed (e.g., IL-1β, TNF-α, and IL-6) in AD patients and compared to controls or other disease groups, such as vascular dementia or cerebrovascular disease, but typically only in cross-sectional or observational studies and not clinical trials [26,27]. We noted a significant decrease (57%) in EGF over the course of the intervention in the Ginkgo Synergy® plus Choline study arm. EGF and its receptor have been classically linked to an over-expression in several tumor cell lines and related to poor prognosis and decreased survival [28]. Additionally, EGF was recently shown to be over-expressed in those with mild cognitive impairment (MCI) or AD compared to healthy controls [29]. Moreover, plasma EGF may be a biomarker for progression to MCI in Parkinson’s patients, as it was correlated with cognitive functioning at baseline and showed predictive capacity over time [30]. Given that neuroinflammation may play a causative role in the pathogenesis of neurodegenerative diseases [31], and many studies have demonstrated mechanistic links among multiple inflammatory pathways in AD [32], finding a way to reduce EGF may help to counteract some of these mechanisms of action. Furthermore, our findings may lend support for the pursuit of anti-EGF therapy, not only for preventing cancer or disrupting carcinogenesis, but for decreasing the negative impact on cognitive functioning.

### Limitations

Several limitations should be noted in the current investigation. We enrolled a predominantly white, non-Hispanic, well-educated sample, so our results may not be generalizable to other populations. In this study, we did not assess dietary intake or physical activity level, so we are unsure how these variables could have affected the results of the study. Nonetheless, subjects were advised not to take any additional dietary supplements, modify their eating habits, or change their physical activity level during the course of the intervention. Our findings may be restricted by the length of the intervention, given that cognitive changes may take longer than 6 months to occur. With the elevated scores in cognitive functioning and a high rate (70%) of existing vitamin/mineral supplement use in our sample, we may also have enrolled “very healthy” individuals who were less sensitive to changes in dietary supplementation. The results of our cytokine and growth factor assays could have been unduly influenced by our participants’ medications that were not initially excluded in our enrollment procedures [33]. For our blood sampling procedures, we utilized all leukocyte cell populations, which could have affected the results of our immune functioning markers [33]. Nonetheless, the Ficoll-Hypaque gradient is easily replicated clinically, does not alter the function of isolated cells, and is routinely used in many settings. Using this methodology, 60-70% of the cells are lymphocytes, while the remaining cells are monocytes and macrophages. The findings of our study are also potentially limited by a small sample size in each study arm.

## Conclusions

The formulae used in the current study were well-tolerated among all subjects, as is typically reported in the literature. Our results show that the Ginkgo Synergy® plus Choline formula is associated with isolated improvements in cognitive and immune functioning. Our findings in cognition are generally consistent with prior work by other investigators using Ginkgo biloba. The OPC Synergy® plus Catalyn® formula (containing antioxidants and A, B, C, and D vitamins) was less effective than Gingko biloba in our study. Its benefit was associated with isolated and short-term improvement in verbal fluency. In summary, our study shows that a high-quality, concentrated Gingko biloba plus choline formula is safe and may offer modest cognitive and immunological benefits, among healthy older adults with no cognitive impairments.

## Abbreviations

TMT: Trail making test; COWA: Controlled oral word association; MMSE: Mini-mental state exam; SCWT: Stroop color and word test; HVLT-R: Hopkins verbal learning test-revised; EGF: Epidermal growth factor; VEGF: Vascular endothelial growth factor.

## Competing interests

JEL, ABM, ET, LC, SL, MH, JD, KG, and JMW have no competing interests to report. JK has received income as a distributor of Standard Process products. EP and DB were employees of Standard Process at the time of study execution. This work was supported by a grant from Standard Process, Inc. Standard Process, Inc. (EP and DB) contributed to the design of the study, but did not implement the study or analyze the data.

## Authors’ contributions

JEL, ABM, ET, LC, SL, MH, JD, KG, JMW, JK, EP and DB contributed to the design of the study. JEL, ABM, ET, LC, SL, MH, JD, KG, JMW, and JK contributed to the collection and analysis of the data. JEL, ET, LC, JK, EP, and DB contributed to the writing of the article. JEL had primary responsibility for final content. All authors read and approved the final manuscript.

## Pre-publication history

The pre-publication history for this paper can be accessed here:

http://www.biomedcentral.com/1472-6882/14/43/prepub
