# Erratum to: A double-blind, randomized clinical trial of dietary supplementation on cognitive and immune functioning in healthy older adults

**DOI:** 10.1186/1472-6882-14-332

**Published:** 2014-09-04

**Authors:** John E Lewis, Angelica B Melillo, Eduard Tiozzo, Lawrence Chen, Susanna Leonard, Mark Howell, Janelle Diaz, Kathy Gonzalez, Judi M Woolger, Janet Konefal, Elaine Paterson, David Barnes

**Affiliations:** Department of Psychiatry & Behavioral Sciences, University of Miami Miller School of Medicine, 1120 NW 14th Street, Miami, FL 33136 USA

## Correction

Since publication of this article [1], the authors have noted three inaccuracies in their report which they would like to amend. The details of these, and the appropriate corrections are detailed below:

1. The Placebo group bars in Figure two (Figure 1 here) were incorrect. The corrected version of this figure can be seen in Figure 1. This correction does not alter the results or interpretation.
2. At the beginning of the discussion, it was stated that the TMT-B time score showed an improvement of 68% after 3 months. This value is incorrect, as the correct value is 18%. While, this change is large it does not change the interpretation or other discussion points.
3. In the Methods section Intervention and Randomization on page 3, the amount of daily vitamin D delivered from the Catalyn should be 416 IU/day, not 312 IU/day. This has no consequences on the results, but improves accuracy of study design reporting.

**Figure 1:**
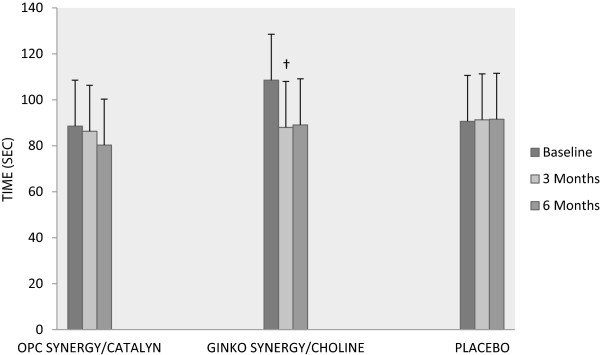
**The CONSORT flowchart.**
